# HCA-DBN: a hill climbing optimized Deep Belief Network for crop yield classification based on kernel weight threshold

**DOI:** 10.3389/frai.2026.1742033

**Published:** 2026-03-12

**Authors:** Prakash Sandhya, B. Venkataramana

**Affiliations:** Department of Mathematics, School of Advanced Sciences, Vellore Institute of Technology, Vellore, India

**Keywords:** Binary classification, Deep Belief Network, field experiment, Hill Climbing Algorithm, maize

## Abstract

Accurate classification of maize yield potential is essential for food security and effective agricultural planning, particularly in regions characterized by environmental variability and socio-economic constraints. This study explores the binary classification of maize kernel weight into low (<25 g) and high (≥25 g) categories, utilizing plant and ear traits collected from an organic maize field experiment in Vellore district, Tamil Nadu (*n* = 160). A Hybrid Cascade – Deep Belief Network (HCA-DBN) is proposed, utilizing the feature extraction capabilities of Deep Belief Networks (DBN) coupled with Hill Climbing Algorithm (HCA) as a lightweight hyperparameter tuning strategy. The model’s performance was benchmarked against standard classifiers including Logistic Regression, Random Forest, XGBoost, Decision Tree, Multi-Layer Perceptron (MLP), and Support Vector Classifier (SVC). The proposed HCA-DBN achieved a peak classification accuracy of 94%, demonstrating its potential to outperform conventional baselines even under small sample conditions. Rigorous validation, including bootstrapping and stratified 10-fold cross-validation, confirmed the statistical stability of the results. While these findings serve as a proof-of-concept given the dataset constraints, this study contributes a methodological benchmark for field-based maize yield classification and provides a scalable framework for future validation on larger, multi-season datasets.

## 1 Introduction

Maize is the cereal crop with highest global production (Statista, 2026), cultivated on nearly 100 million hectares across 125 countries, and ranks among the top three staple crops in over 75 nations. Its leading production status reflects maize’s adaptability to diverse agroecological conditions and its central role in global food and feed systems. Although roughly 80% of maize output is used as livestock feed, its direct consumption by humans has been increasing in both developing and developed economies. In India, maize ranks fourth in area and seventh in production, accounting for approximately 4% of the global maize area and 2% of world output. These facts underline the critical need to improve maize productivity through region-specific hybrids and varieties, motivating accurate pre-harvest yield forecasting to support food security and farm management under climate variability. This study is based on a field dataset (*n* = 160 tagged plants) from an organic maize trial conducted in the Vellore district, Tamil Nadu. While limited in size, such datasets are common in resource-constrained agricultural research and provide a useful testbed for evaluating model feasibility.

A broad range of regression and machine learning approaches has been applied to crop yield prediction. Interaction regression models incorporating soil, weather and crop management factors have been developed to predict maize yield; however, such methods often struggled to attain global optima and become computationally expensive when higher order interactions were considered (Ansarifar et al., 2021). Crop simulation tools (e.g., ABSOLUT v1.2) have been applied to estimate yields from meteorological inputs, demonstrating that optimized regression approaches can be competitive with machine learning techniques within defined forecasting scenarios. However, their performance typically declines under greater climate variability and over longer aggregation periods (Conradt, 2022). More recently, ensemble methods such as XGBoost have been widely adopted for yield forecasting. XGBoost has been paired with interpretability tools like SHAP and tree-based adaptations to enhance both accuracy and transparency in multi-crop predictions (Huber et al., 2022; Mariadass et al., 2022; Noorunnahar et al., 2023; Kalichkin et al., 2021; Borse and Agnihotri, 2024). Random Forest and Light Use Efficiency (LUE) models have also been used to identify key predictors, with performance improving further when combined with random search or other optimization schemes (Dhillon et al., 2023; Padma and Sinha, 2023).

Classification and probabilistic methods have supplemented regression frameworks in several studies. Naïve Bayes classifiers have been applied for decision support in crop management due to their simplicity and robustness (Chavan et al., 2022; Patil et al., 2023). Support Vector Regression (SVR) and Support Vector Classifiers (SVC) have been utilized to address prediction errors in grain yield, often combined with feature selection and optimization methods such as grid search and genetic algorithms (Wang et al., 2023; Shafiee et al., 2021; Rajakumaran et al., 2024). Ensemble strategies, such as bagging, stacking and boosting, have consistently improved predictive performance when integrated with algorithms like XGBoost (Tiwari et al., n.d.; Sankareswari and Sujatha, 2023). Furthermore, the classification of yields into categorical tiers (e.g., low, medium, high) based on meteorological summaries has been explored, with multi-class models sometimes outperforming binary approaches in specific contexts (Sharma et al., 2024). To address issues of multicollinearity and model interpretability, regularization and feature selection methods have been widely adopted. Binary, ordinal and multinomial Logistic Regression models have been applied where ordinal target variables were appropriate (Kumari et al., 2016; Kumari, 2012). LASSO and Ridge regularization often outperform unregularized regression by selecting influential predictors and stabilizing coefficient estimates (Singh et al., 2019; Saranya and Sathappan, n.d.). The Elastic Net (ELNET) approach, which combines the penalties of Ridge and LASSO, has further improved prediction in settings with correlated features (Lakshmanarao et al., 2023; Li et al., 2021).

Deep learning methods have increasingly been explored for yield estimation because of their capacity to model complex, nonlinear relationships among predictors. Multi-Layer Perceptrons (MLPs) have been applied to both remote sensing and field-level datasets, demonstrating performance comparable to classical regression in several studies (Tripathi et al., 2022; Geetha and Elizabeth Shanthi, 2020). While deep networks effectively capture environment – cultivar interactions, they typically require substantial labeled data, careful architecture design, and tuned activation functions - resources that are not always available in agricultural experiments (Duarte De Souza et al., 2023; Feng et al., 2017). Deep Belief Networks (DBN), constructed by stacking Restricted Boltzmann Machines (RBM), present an alternative that can learn hierarchical feature representations while reducing dependence on labeled data. Iterative DBN variants and hybrid approaches that combine dimensionality reduction (e.g., PCA) with DBNs have been proposed to improve efficiency and classification performance, though challenges with parameter selection, computational cost and overfitting persist (Zambra et al., 2023; Kabir et al., 2023; Pinaya et al., 2016).

While deep learning methods are often associated with large datasets, prior work has shown that DBNs can be trained effectively on small-to-medium datasets when combined with appropriate pre-training strategies. For example, recent studies demonstrated that DBNs outperform compact models on limited protein structure datasets (Bondarenko and Borisov, 2013) and achieve competitive performance on classification tasks with modest sample sizes (Rashid et al., 2023). Additionally, applications in clinical prediction with tens to low hundreds of samples have been published, suggesting that DBNs can generalize even with constrained data (Li et al., 2022). Prior research in optimizing network structure for small sample sizes further supports the importance of architecture search in such regimes (D’souza et al., 2020).

Hyperparameter optimization therefore remains a central obstacle for DBN deployment in yield classification tasks. Metaheuristic and local search algorithms have been applied to address this, and simple but effective heuristics such as the Hill Climbing Algorithm (HCA) have been explored for tuning network structure and other parameters (Xie and Li, 2021). Unlike Bayesian or evolutionary optimization methods, HCA is lightweight and computationally inexpensive, making it suitable for exploratory analysis on small, field-collected datasets. Rather than preselecting a reduced subset of predictors, using the complete set of measured plant and ear traits allows the model to leverage all available phenotypic information and avoids the risk of excluding potentially informative variables. In this study, we adopt that approach and treat kernel weight as a binary classification target (low: <25 g, high: ≥25 g). This threshold was selected to reflect a practical cut-off for field evaluation, as ears below 25 g in this trial typically exhibited incomplete seed filling. Additionally, as this threshold approximates the dataset median, it ensures class balance – a critical factor for preventing bias during model optimization.

Building on these observations, the present study proposes a Hybrid Cascade – Deep Belief Network (HCA-DBN) in which HCA optimizes key DBN hyperparameters (number of neurons per layer, activation functions, optimizer and learning rate). The HCA-DBN is evaluated against standard classifiers including Logistic Regression, Random Forest, XGBoost, Decision Tree, MLP and SVC using field-collected data. The main contributions of the study are as follows:

- Development of the HCA-DBN hybrid classifier tailored for maize kernel weight classification.
- The retention of the complete array of measured plant and ear traits as predictors, ensuring the avoidance of pre-emptive feature exclusion.
- Integration of hill climbing into the DBN optimization process to automate hyperparameter selection.
- Empirical validation on field-collected maize data and comparative evaluation against established classifiers.

The primary innovation lies in demonstrating the feasibility of combining DBNs with lightweight optimization on small field-collected maize datasets. The resulting proof-of-concept establishes a methodological benchmark that can be extended in future studies using larger multi-season datasets.

## 2 Study area and data acquisition

### 2.1 Experimental site and design

**Figure 1:**
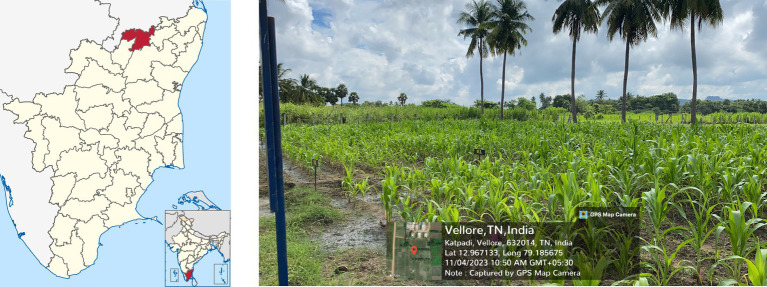
The experimental area’s location – Sevur (Vellore).

The field experiment was conducted at the Sevur farm of the Vellore Institute of Technology (VIT), Vellore, Tamil Nadu [12.97°N, 79.19°E; 213 m above Mean Sea Level (MSL)] (Radhakrishnan et al., 2024). The experimental location is illustrated in Figure 1. The region is characterized by a semi-arid climate with a mean annual rainfall of approximately 971 mm (Agriculture, 2026). To evaluate yield stability under sub-optimal environmental conditions, the crop was sown during the off-season (late kharif) on clay-textured soil. A Randomized Block Design (RBD) with four replications was adopted to control field variability. Eight maize genotypes were evaluated, comprising four novel hybrids developed by the Indian Institute of Maize Research (IIMR), Punjab, and four local farmer-preferred varieties.

The field experiment was initially established to cover two cropping seasons. However, data acquisition for this study was restricted to the first season. The second season crop was excluded from the final dataset due to severe environmental confounding factors, including prolonged waterlogging caused by heavy rainfall on clayey soil and extensive vertebrate pest damage (wild boar and parrot infestation), which resulted in an insufficient sample size for robust statistical analysis. Consequently, the final dataset contains 160 observations derived from five randomly tagged plants per plot in the first season. To ensure data consistency and avoid sampling bias, all biometric and yield measurements were strictly restricted to those tagged plants.

### 2.2 Data collection and feature description

Maize breeding efforts typically prioritize traits that directly contribute to yield, such as ear dimension and vegetative robustness. In this study, kernel weight (KW) was selected as the target variable for classification. The predictor variables comprised a comprehensive set of agronomic and physiological traits. These included morphological traits such as plant height (PH), number of leaves (NOL), ear weight (EW), ear length (EL), and kernels per row (KR), alongside physiological metrics.

Canopy temperature (CT) was recorded using a FLIR E8 infrared imaging camera (FLIR Systems), and chlorophyll content (Ch) was measured using a V-TECH chlorophyll meter. Measurements for CT and Ch were recorded at five distinct growth stages (denoted as CT1–CT5 and Ch1–Ch5). The complete list of measured traits, their abbreviations, and respective units is presented in Table 1.

**Table 1:** Parameter measurement type and their units.

Attributes	Code	Units	Measurement type
Vegetative attributes			
Plant number	PN	-	Tagged plant count
Plant height	PH	mm	From crown to base
Number of leaves	NOL	count	Manually counted
Number of nodes	NON	count	Manually counted
Number of cobs	NOC	count	Manually counted
Number of tassels	NOT	count	Manually counted
Plant height at cob	PHC	mm	Base to ear node
Leaf length	LL	mm	Length of flag leaf
Leaf breadth	LB	mm	Width of flag leaf
Yield attributes			
Ear weight with sheath	EWS	g	Weighing scale
Ear weight without sheath	EWOS	g	Weighing scale
Ear length	EL	mm	Distal to proximal end
Ear width	EW	mm	Width of dehusked ear
Rows per ear	RE	count	Manually counted
Kernels per row	KR	count	Manually counted
Kernel weight	KW	g	Weighing scale
Canopy temperature	CT	°C	FLIR E8 thermal camera (45° view)
Chlorophyll content	Ch	CCI units	Chlorophyll (7:00–9:00 a.m.)

### 2.3 Data pre-processing

**Figure 2:**
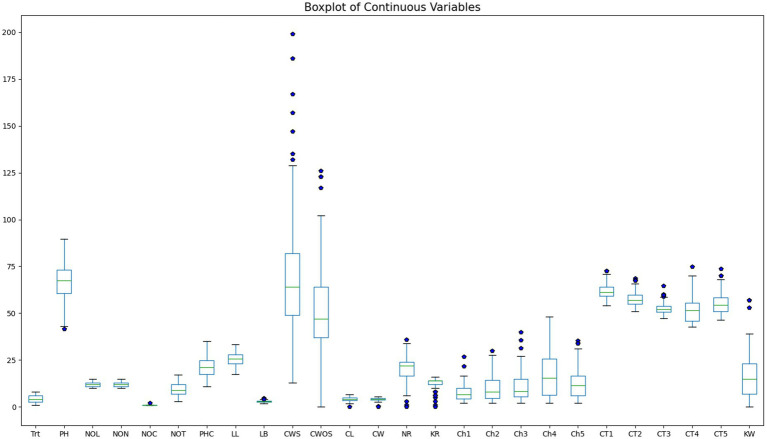
Boxplot of continuous variables.

The dataset comprised 160 observations with 26 features. Pre-processing involved handling missing values, normalization and standardization to prepare the data for classification. One observation was identified with missing values due to pest infestation on a tagged plant and was subsequently removed from the dataset. Descriptive statistics for all 26 measured traits are summarized in Table 2. The distribution of features was assessed using boxplots (Figure 2), Q–Q plots, histograms, and statistical normality tests (Anderson-Darling, D’Agostino K^2^, Kolmogorov Smirnov, Jarque Bera, Shapiro Wilk), which revealed skewness in several variables. To address this, various transformation techniques, including logarithmic, square root, reciprocal, Box Cox, and Yeo Johnson methods, were evaluated.

**Table 2:** Descriptive statistics.

Attributes	Count	Average	SD	Min	Q1	Q2	Q3	Max
Treatment	160	4.50	2.30	1.00	2.75	4.50	6.25	8.00
PN	160	10.50	5.78	1.00	5.75	10.50	15.25	20.00
PH	160	66.90	8.75	41.50	60.88	67.50	73.05	89.50
NOL	160	11.98	1.14	10.00	11.00	12.00	13.00	15.00
NON	160	12.27	1.20	10.00	11.00	12.00	13.00	15.00
NOC	160	1.01	0.08	1.00	1.00	1.00	1.00	2.00
NOT	160	9.55	3.36	3.00	7.00	9.00	12.00	17.00
PHC	160	21.43	4.64	11.00	17.58	21.00	24.70	35.00
LL	160	25.61	3.51	17.50	23.15	25.80	27.70	33.20
LB	160	2.95	0.44	1.90	2.68	2.90	3.20	4.50
EWS	160	69.13	30.79	13.00	49.00	64.00	82.00	199.00
EWOS	160	51.87	23.04	0.00	37.00	47.50	64.00	126.00
EL	160	4.14	0.93	0.00	3.50	4.20	4.80	6.50
EW	160	4.42	4.02	0.00	3.68	4.20	4.60	54.00
RE	160	20.83	6.52	0.00	16.75	22.00	24.25	36.00
KR	160	12.71	2.63	0.00	12.00	14.00	14.00	16.00
KW (yield)	160	16.23	11.41	0.00	7.00	15.50	23.00	57.00
Ch1	160	7.60	4.13	2.10	4.30	6.75	10.13	26.90
Ch2	160	9.84	6.61	2.00	4.48	7.95	14.23	29.80
Ch3	160	10.85	7.13	2.00	5.40	8.30	15.00	39.90
Ch4	160	17.34	11.53	2.10	6.28	15.70	25.93	48.10
Ch5	159	12.20	7.37	2.10	6.15	11.50	16.50	35.40
CT1	160	61.88	4.01	54.20	58.98	61.18	64.03	72.55
CT2	160	57.55	3.67	50.88	54.89	56.95	59.80	68.60
CT3	160	52.57	2.62	47.15	50.79	52.22	53.95	64.50
CT4	160	51.47	6.13	42.15	45.60	51.45	55.48	74.80
CT5	160	55.55	5.72	46.30	51.05	54.50	58.23	73.80

**Figure 3:**
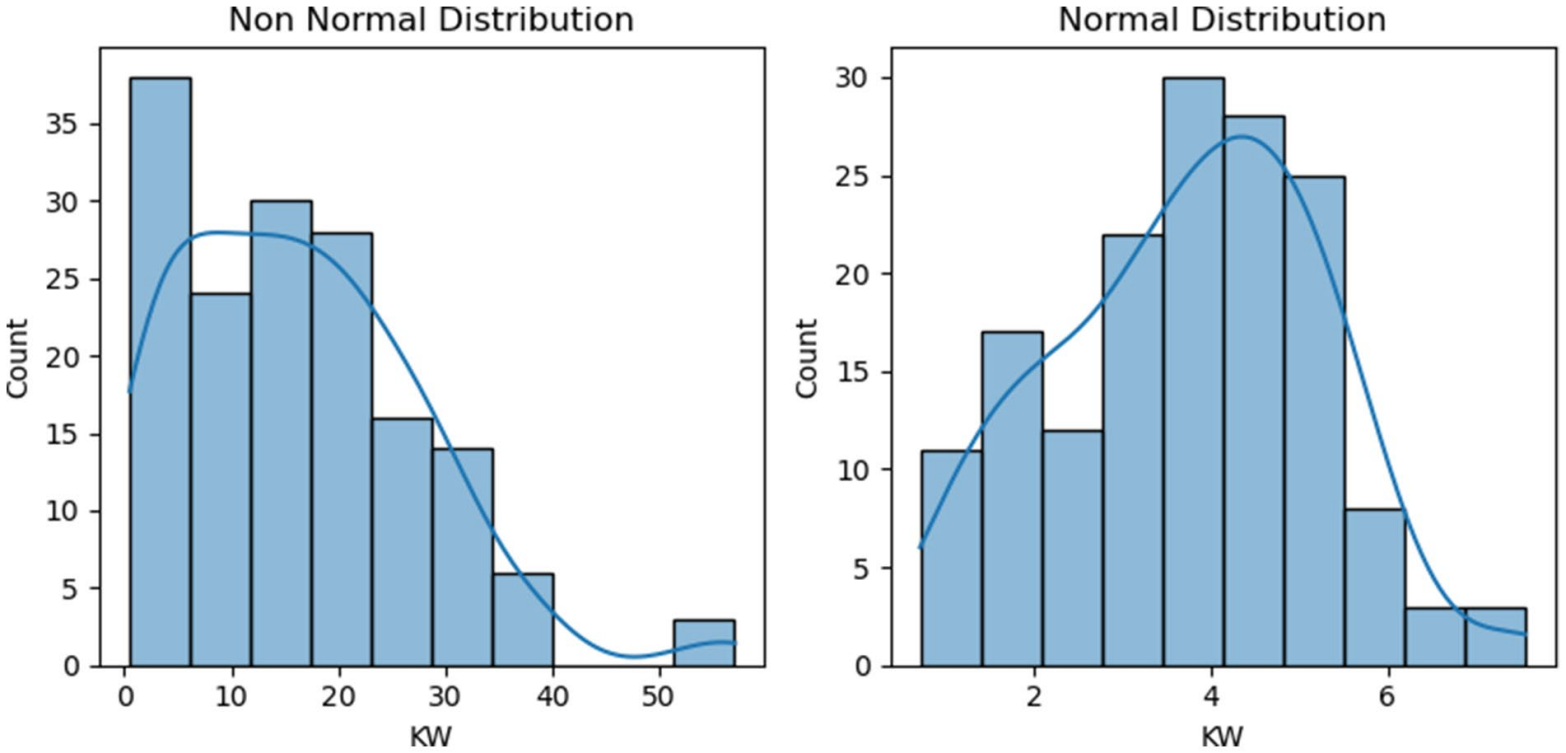
Distribution of kernel weight prior and post square root transformation.

Comparative analysis demonstrated that the square root transformation yielded the superior improvement in normality metrics across the board, as illustrated for kernel weight in Figure 3. Consequently, the square root transformation was applied uniformly to all continuous predictor variables to standardise the statistical pre-processing. While the transformation satisfied the assumptions for statistical testing, the feature set for DBN was subsequently subjected to Min-Max Scaling (mapping values to [0,1]). This ensured that all inputs were aligned with the active region of the network’s activation functions, facilitating efficient and stable gradient descent.

**Figure 4:**
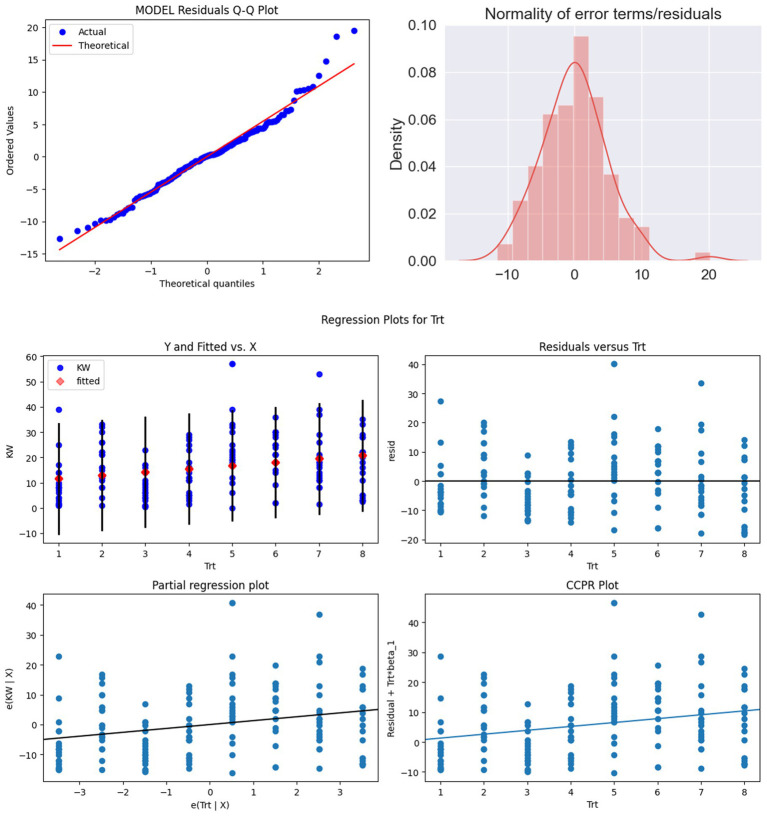
Normality of residuals depicted by Q–Q plot and distribution plot.

Model assumptions were assessed prior to analysis. Linearity between dependent and independent variables was verified using scatter plots, while homoscedasticity was confirmed using White’s and Goldfeld Quandt tests. Multicollinearity was evaluated using variance inflation factor (VIF), which revealed high correlation between ear weight with sheath (EWS) and ear weight without sheath (EWOS), and moderate correlations among PH, RE, and EW (Table 3). The normality of residuals was further confirmed through Jarque Bera tests and Q–Q plots (Figure 4), while residual versus fitted plots were examined to rule out underlying non-linear trends.

**Table 3:** Variance inflation factor (VIF) of predictor variables.

Variable	VIF	Variable	VIF
Treatment	3.22	RE	5.12
PH	5.08	KR	2.54
NOL	2.67	Ch1	1.17
NON	3.32	Ch2	1.29
NOC	1.28	Ch3	1.85
NOT	1.57	Ch4	1.86
PHC	3.25	Ch5	1.31
LL	3.44	CT1	1.71
LB	2.39	CT2	2.07
EWS	13.07	CT3	1.58
EWOS	15.77	CT4	1.61
EL	4.35	CT5	1.43
EW	6.08		

### 2.4 Data splitting

Given the limited dataset size (*n* = 160), all results are interpreted as preliminary and data specific, highlighting the need for future validation on larger, multi-season datasets. To evaluate model robustness, the dataset was split into training and testing subsets using four different ratios (60:40, 70:30, 75:25 and 80:20) a stratification strategy that allows for the assessment of model performance stability across varying training sample sizes.

### 2.5 Performance metrics and model diagnostics

The proposed model was evaluated based on its ability to correctly categorize maize kernel weight into two classes: low yield (KW < 25) and high yield (KW ≥ 25). Standard classification metrics were employed:

- Accuracy: Proportion of correctly classified samples across both classes.

$$\text{Accuracy}=\frac{N_{\mathrm{low},\text{correct}}+N_{\text{high},\text{correct}}}{N_{\text{total}}}$$

- Error rate: The proportion of misclassified samples.

$$\text{Error Rate}=\frac{N_{\mathrm{low},\text{misclassified}}+N_{\text{high},\text{misclassified}}}{N_{\text{total}}}$$

- Recall (sensitivity): The ability of the model to correctly identify samples in each class.

$$\text{Recal}l_{\mathrm{low}}=\frac{N_{\mathrm{low},\text{correct}}}{N_{\mathrm{low},\text{total}}}\;\;\;\text{Recal}l_{\text{high}}=\frac{N_{\text{high},\text{correct}}}{N_{\text{high},\text{total}}}$$

- Precision: The proportion of correctly predicted samples within each predicted class.

$$\text{Precisio}n_{\mathrm{low}}=\frac{N_{\mathrm{low},\text{correct}}}{N_{\text{predicted}\;\mathrm{low}}}\;\;\;\text{Precisio}n_{\text{high}}=\frac{N_{\text{high},\text{correct}}}{N_{\text{predicted high}}}$$

- F1-score: Harmonic mean of recall and precision for each class.

$$F1_{\mathrm{low}}=2.\;\;\frac{\text{Precisio}n_{\mathrm{low}}.\text{Recal}l_{\mathrm{low}}\;}{\text{Precisio}n_{\mathrm{low}}+\text{Recal}l_{\mathrm{low}}}\;F1_{\text{high}}=2.\;\;\frac{\text{Precisio}n_{\text{high}}.\text{Recal}l_{\text{high}}\;}{\text{Precisio}n_{\text{high}}+\text{Recal}l_{\text{high}}}$$

Additionally, training and validation loss curves were analysed to visualize the learning process. Decreasing training loss indicates improvement in predictive ability, while discrepancies between training and validation loss signal potential overfitting. These metrics provide a comprehensive evaluation of the model’s ability to correctly classify individual maize ears into low and high kernel weight categories.

## 3 Model configuration and fine-tuning

### 3.1 Model investigation

Initial experiments were conducted using various classification models to evaluate their suitability for classifying maize kernel weight (KW) categories. Baseline models, including Logistic Regression and linear Support Vector Machines (SVM), were evaluated to establish a reference performance. However, these linear classifiers were unable to capture the complex non-linear interactions between plant morphology, ear traits, canopy temperature, and chlorophyll features, resulting in limited classification accuracy and a failure to fully represent underlying biological patterns.

Ensemble-based methods, such as Random Forest and Gradient Boosting Machines (GBM) were subsequently explored. While these models successfully captured intricate feature interactions and produced higher training accuracy, their sensitivity to small-scale variations and noise in the limited dataset (*n* = 160) made them prone to overfitting, resulting in reduced generalization to unseen test samples. To overcome the shortcomings of conventional classifiers, this study introduces a Hybrid Cascade – Deep Belief Network (HCA-DBN) model. By integrating the hierarchical feature learning capacity of DBNs with the efficiency of heuristic optimization, the HCA-DBN achieves a balance between accuracy and generalization. Comparative evaluations demonstrated that the optimized HCA-DBN consistently outperformed conventional classifiers, achieving robust and stable performance in maize yield classification.

### 3.2 Deep Belief Network

**Figure 5:**
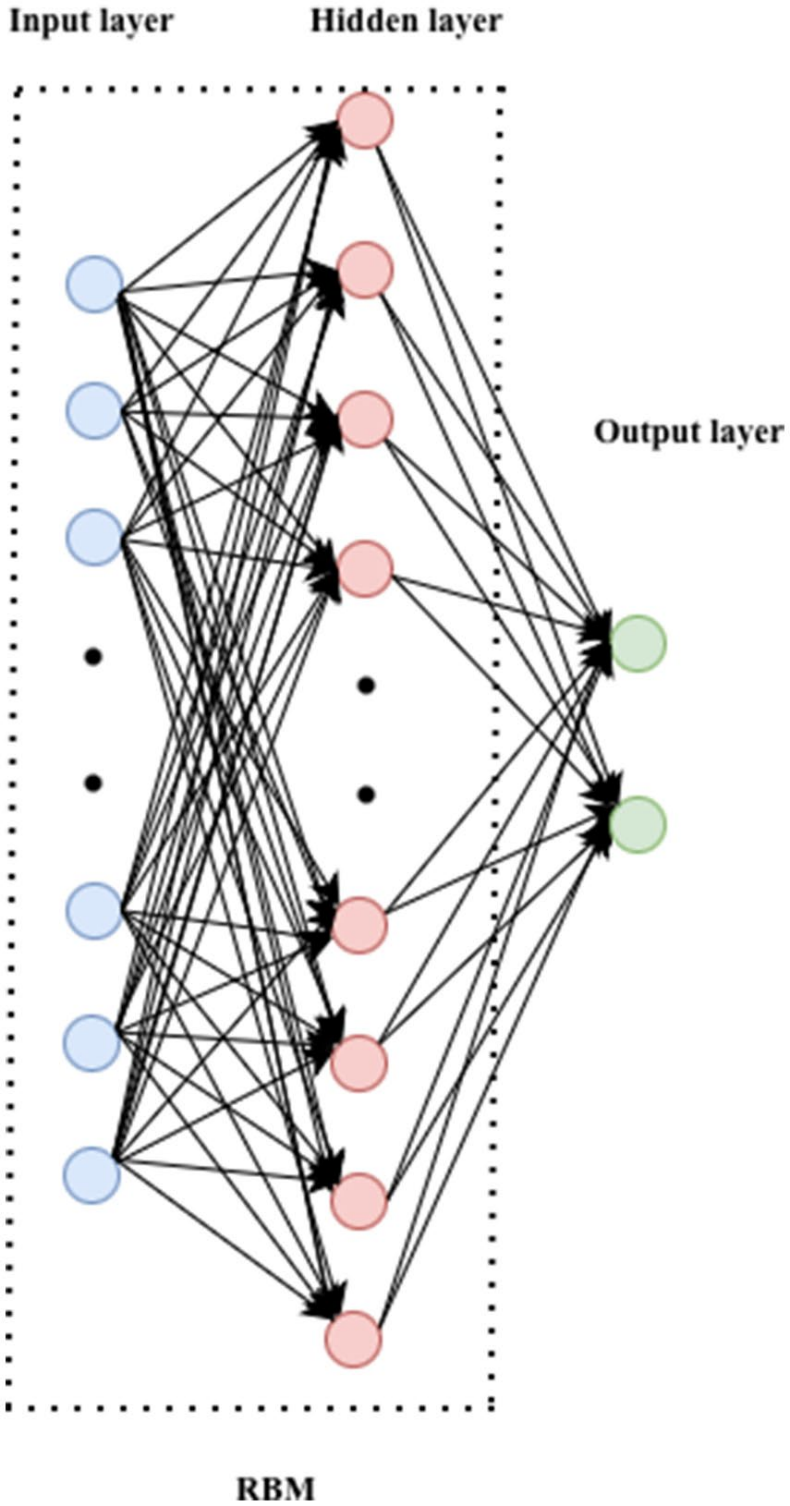
DBN architecture.

A Deep Belief Network is a generative probabilistic model composed of multiple stacked Restricted Boltzmann Machines (RBMs). Each RBM learns latent feature representations through unsupervised training, where the outputs from one RBM layer serve as inputs to the next. This hierarchical architecture enables DBNs to capture complex patterns in high dimensional data (Figure 5).

For a standard RBM, visible units $v=\{v_{1},v_{2},\ldots ,v_{m}\}$ represent input features, while hidden units $h=\{h_{1},h_{2},\ldots ,h_{n}\}$ capture latent representations. The energy function E(v,h) for a given configuration is defined as:


$$E(v,h|\theta )=-\sum_{i=1}^{m}a_{i}v_{i}-\sum_{j=1}^{n}b_{j}h_{j}-\sum_{i=1}^{m}\sum_{j=1}^{n}v_{i}w_{\mathrm{ij}}h_{j}$$

where $w_{\mathrm{ij}}$ denotes the weight between visible unit i and hidden unit j , while $a_{i}$ and $b_{j}$ represent the bias terms for visible and hidden layers, respectively. The model parameters are θ = { $w_{\mathrm{ij}}$ , $a_{i}$ , $b_{j}$ }. The joint probability distribution of v and h is given by the Boltzmann distribution:


$$P(v,h\;|\theta )=\frac{e^{-E(v,h\;|\theta )}}{Z^{\theta }}$$

where Z is the partition function (normalization factor):


$$Z^{\theta }=\sum_{v,h}e^{-E(v,h\;|\theta )}$$

Since there are no intra-layer connections in an RBM, the conditional probabilities for activation are given by:


$$P(h_{j}=1|v,\theta )=\sigma (b_{j}+\sum_{i=1}^{m}v_{i}w_{\mathrm{ij}})$$

$$P(v_{i}=1|h,\theta )=\sigma (a_{i}+\sum_{i=1}^{n}h_{j}w_{\mathrm{ij}})$$

where σ(·) is the sigmoid activation function. RBM parameters are optimized by maximizing the log-likelihood function. However, because exact likelihood computation is intractable, approximate methods such as Contrastive Divergence (CD-K) are employed (Romero et al., 2019). This unsupervised pre-training enables the DBN to learn robust hierarchical feature representation before supervised fine tuning.

### 3.3 Hill Climbing Algorithm (HCA)

The Hill Climbing Algorithm (HCA) is a heuristic optimization approach that iteratively improves a candidate solution by evaluating neighbouring configurations and selecting the one that maximizes performance. Starting from a random initialization, HCA searches for optimal hyperparameters until convergence at a local optimum (Lu et al., 2023). In the context of this study, HCA is employed to optimize critical DBN hyperparameters, specifically: number of hidden layers, neurons per layer, dropout rate, activation functions, learning rate, and batch size. Unlike computationally expensive global search methods (e.g., Genetic Algorithms), HCA is lightweight and efficient, making it particularly suitable for optimizing deep networks on smaller datasets where rapid convergence is required.

### 3.4 HCA optimized DBN classification model

The integration of DBN and HCA proceeds in a cascade framework comprising four major steps:

1. Structure definition: The input data vector X is processed through stacked RBM layers.
2. Unsupervised pre-training: RBMs are trained layer-by-layer using Contrastive Divergence to extract hierarchical features.
3. HCA optimization: Hyperparameters are iteratively tuned. For each iteration, the algorithm generates neighbouring architectures (e.g., adding a neuron, changing the learning rate), evaluates their validation accuracy, and moves to the better configuration.
4. Supervised fine tuning: The pre-trained and optimized DBN is fine-tuned using backpropagation with labeled maize KW data (Low vs. High) to minimize classification error.

This cascade-optimized architecture ensures that the model captures relevant biological relationships while maintaining generalization to unseen samples. The detailed computational procedure of the proposed HCA-DBN framework is summarized in Algorithm 1.

**ALGORITHM 1:**
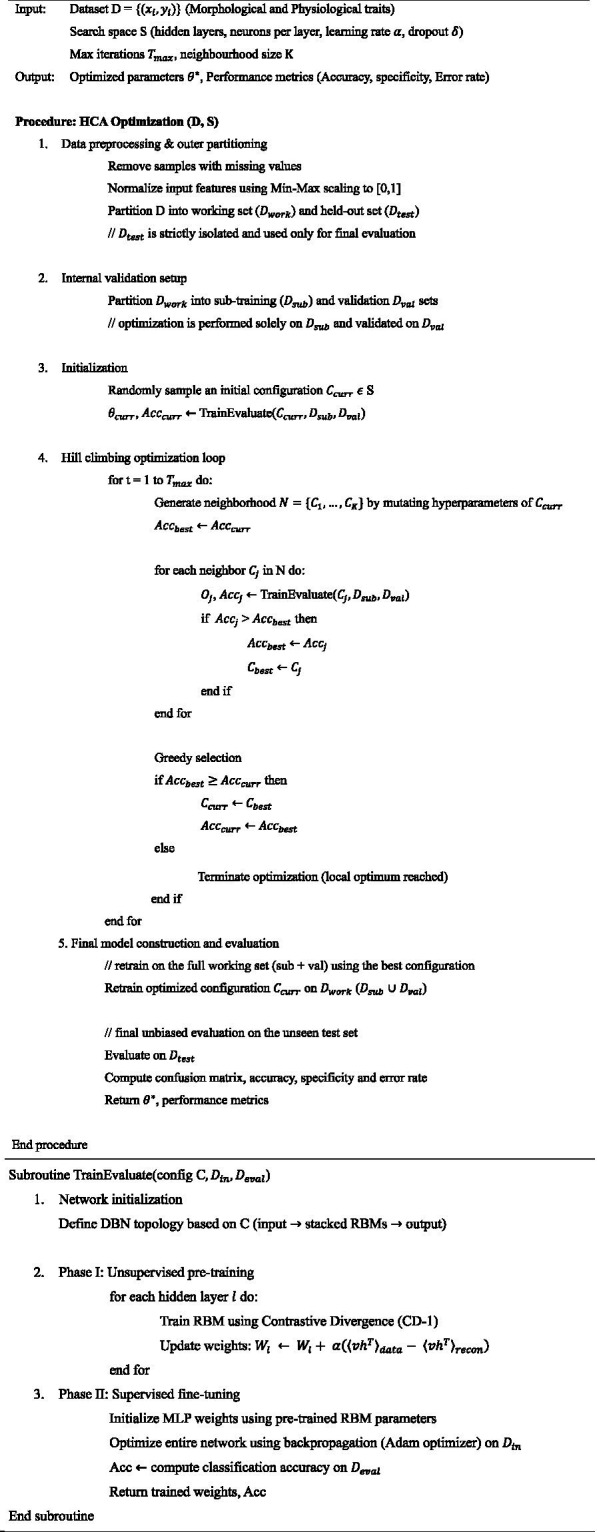
Hill climbing optimized Deep Belief Network (HCA-DBN).

### 3.5 Hyper-parameter tuning and final model

**Figure 6:**
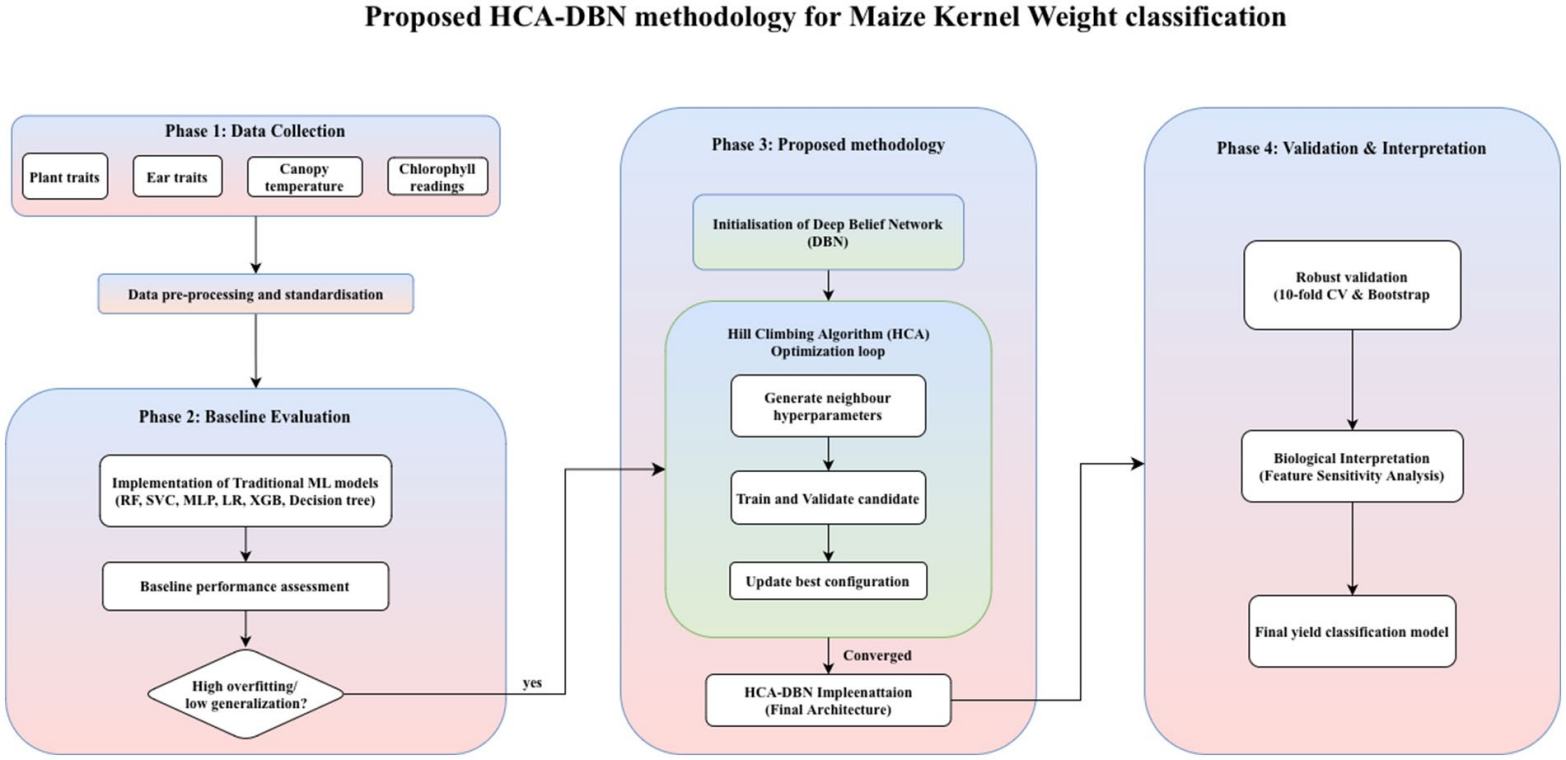
Flowchart of the proposed HCA-DBN model.

This study optimized the DBN model using the Hill-Climbing Algorithm (HCA) to enhance classification performance for kernel weight prediction. While DBNs provide a strong framework for hierarchical feature learning, their performance depends heavily on the choice of hyperparameters. The workflow of the proposed HCA-DBN framework is presented in Figure 6. Parameters including the number of hidden neurons, batch size, dropout rate, activation function, optimizer, and epochs were systematically tuned.

- Hidden neurons: Tested within the range of [2–500] to identify the optimal network depth and capacity.
- Batch size: Values of 2, 4, 8, 16, 32 and 64 were compared to balance convergence stability and training efficiency.
- Dropout rate: Rates from 0.20 to 0.50 were explored to improve generalization.
- Optimizer: Multiple optimizers including Nadam, RMSProp, Adam and SGD were tested to achieve stable and efficient convergence.
- Activation functions: Both linear and nonlinear activations such as Sigmoid, ReLU, LeakyReLU and Swish were assessed.
- Epochs: Training was conducted across 50 to 200 epochs to determine the optimal convergence point.

The combination of hierarchical feature representation from DBNs, progressive complexity control from cascade learning, and adaptive fine-tuning with HCA resulted in a robust model architecture. To strictly evaluate the model’s generalization capability and address the constraints of the limited dataset size, the final optimized HCA-DBN model was subjected to two rigorous validation scenarios beyond standard train-test splitting:

1. Stratified 10-fold cross-validation: Utilized to ensure every sample was used for both training and testing, minimizing the bias associated with fixed splits.
2. Bootstrap validation: A bootstrap analysis (200 iterations) was conducted to estimate the confidence intervals of the model’s accuracy and verify statistical stability.

## 4 Results and discussion

The objective of this study was to classify maize kernel weight into yield categories using plant and ear traits collected from an organic maize field trial in Vellore district, Tamil Nadu. Individual ear kernel weights were measured after shelling. Table 4 presents the total kernel weight per maize variety to indicate overall varietal productivity. Individual kernel weights ranged from 0 to 57 g, and a threshold of 25 g was chosen to define a balanced binary classification framework.

**Table 4:** Kernel weight of treatments.

Treatment	Genotype name	Total KW (g)
5	**Shivani KSMH 1980**	**477.00**
6	Pioneer 30B5307	426.00
7	RMH 3414	397.50
2	IMH 223	349.00
8	RMH 3591	323.50
4	IMHSB 19 KB-2	277.50
1	IMH 222	183.00
3	IMH 224	163.00

**Figure 7:**
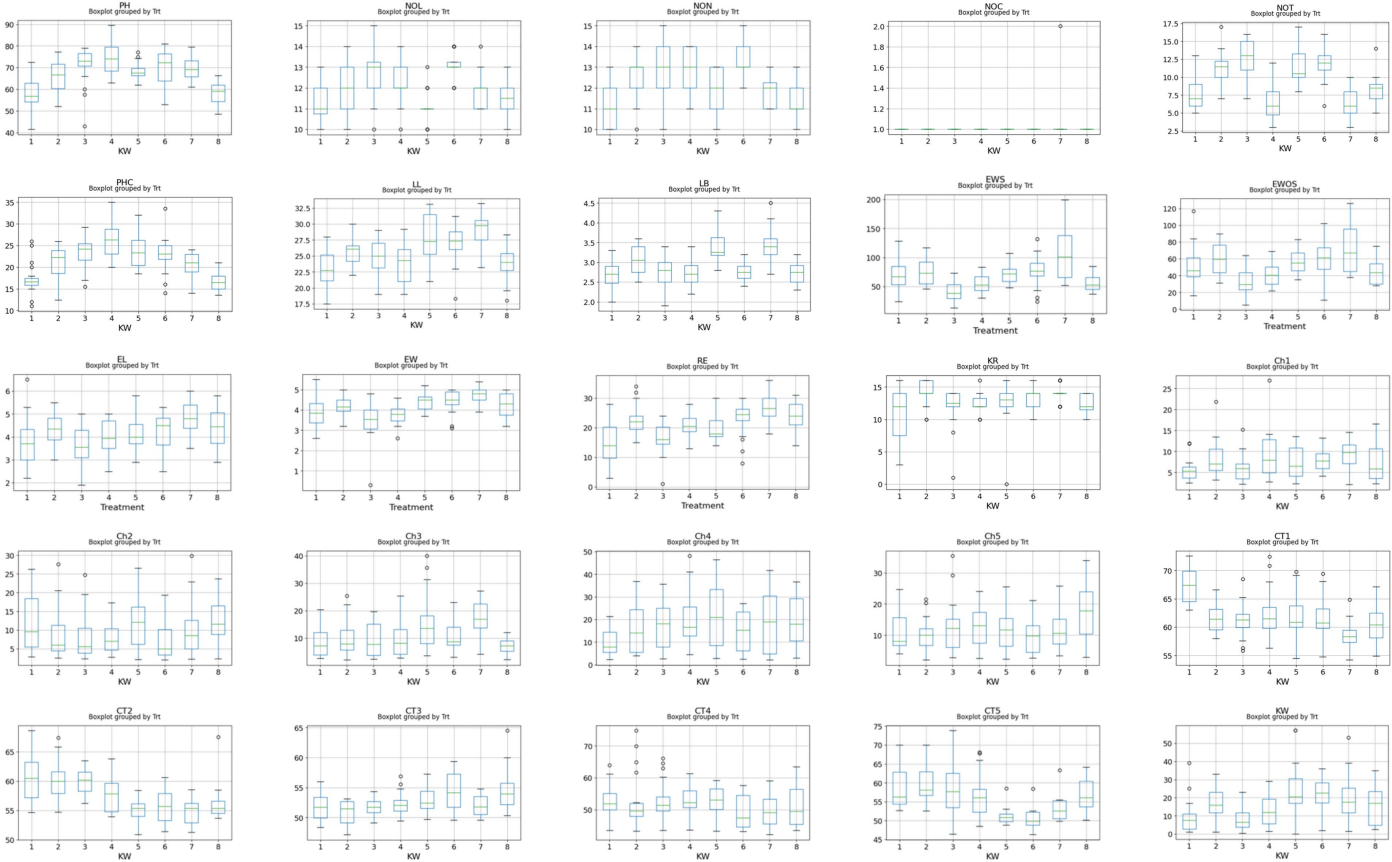
Box plot of each feature grouped by treatment.

Analysis of varietal performance revealed that variety 5 (Shivani KSMH 1980, 477 g) exhibited the highest total kernel weight, followed by variety 6 (426 g) and variety 7 (397.5 g), while variety 3 (163 g) and variety 1 (183 g) recorded the lowest productivity. To visualize the distribution of plant and ear traits across varieties, boxplots were generated for each feature (Figure 7). The plots highlight clear varietal differences in plant height, leaf number, ear weight, and kernel rows, demonstrating the inherent phenotypic variability crucial for predicting individual ear kernel weight.

### 4.1 Baseline classifier performance

Initial evaluation using conventional classifiers (Table 5) revealed significant performance disparities. Linear models like Logistic Regression and SVC struggled to separate the classes, achieving low testing accuracies of 0.56 and 0.59 respectively, indicating that the relationship between maize traits and yield is highly non-linear. Among the conventional models, Random Forest achieved the highest baseline performance with a testing accuracy of 0.87. However, it exhibited clear signs of overfitting, with a perfect training accuracy of 1.00 dropping to 0.87 on the test set. Similarly, XGBoost and Decision Tree models achieved 1.00 training accuracy but failed to generalize effectively, dropping to 0.75 and 0.69 on testing data. Multi-Layer Perceptron (MLP), while avoiding severe overfitting (Train 0.76, Test 0.78), failed to achieve competitive predictive power. This consistent struggle across baselines to balance high accuracy with generalization highlighted the need for the proposed HCA-DBN framework.

**Table 5:** Traditional forecasting models implemented to the data and their accuracy.

Model	Nature	Accuracy (test)	Accuracy (train)
Random Forest	Non-linear	0.87	1.00
Logistic Regression	Linear	0.56	0.84
Support Vector Classifier	Non-linear	0.59	0.89
Multi-Layer Perceptron	Non-linear	0.78	0.76
XGBoost	Non-linear	0.75	1.00
Decision Tree	Non-linear	0.69	1.00
Deep Belief Network (unoptimized)	Non-linear	0.89	0.92

#### 4.1.1 Baseline model tuning

To ensure a fair comparison with the DBN, the baseline classifiers were optimized using a systematic grid search strategy rather than relying on default parameters. This process ensured that the performance gaps observed were due to model architecture limitations rather than poor initialization. The specific hyperparameter settings selected for each baseline model are detailed below.

- Random Forest and Decision Tree: Optimized for maximum depth (10-None) and minimum samples split to control the trade-off between capturing complex patterns and overfitting. For Random Forest, the number of estimators was tuned between 50 and 200.
- XGBoost: Key boosting parameters including learning rate (0.01–0.2), number of estimators, and maximum depth were adjusted to prevent the model from memorizing the training data.
- SVM and Logistic Regression: For the SVM, the kernel type (RBF vs. Linear) and regularization parameter ∁ were tuned. Similarly, Logistic Regression was optimized for different solvers and ∁ values to handle the high-dimensional feature space.
- Multi-Layer Perceptron (MLP): A standard feed-forward network was tested with varying hidden layer architectures (single vs. dual layers) and activation functions.

### 4.2 HAC-DBN optimization and performance

The Deep Belief Network (DBN) was selected for further optimization due to its ability to learn hierarchical feature representations and mitigate overfitting through layer-wise unsupervised pre-training followed by supervised fine-tuning (Vinyals and Ravuri, n.d.). DBNs are particularly well suited for structured datasets with limited samples (Xie and Li, 2021), allowing them to capture subtle interactions among plant and ear traits that classical ML models and standard MLP may miss.

The HCA optimization process specifically constrained to balance classification accuracy with model complexity. While the search space included deep multi-layer configurations (up to 500 units), the algorithm converged on a compact topology of Rashid et al. (2023) hidden units rather than a deep network. This automatic selection validates that smaller, denser representations are more effective for the 26-feature input space, avoiding the vanishing gradient and overfitting problems associated with over-parameterized networks.

**Figure 8:**
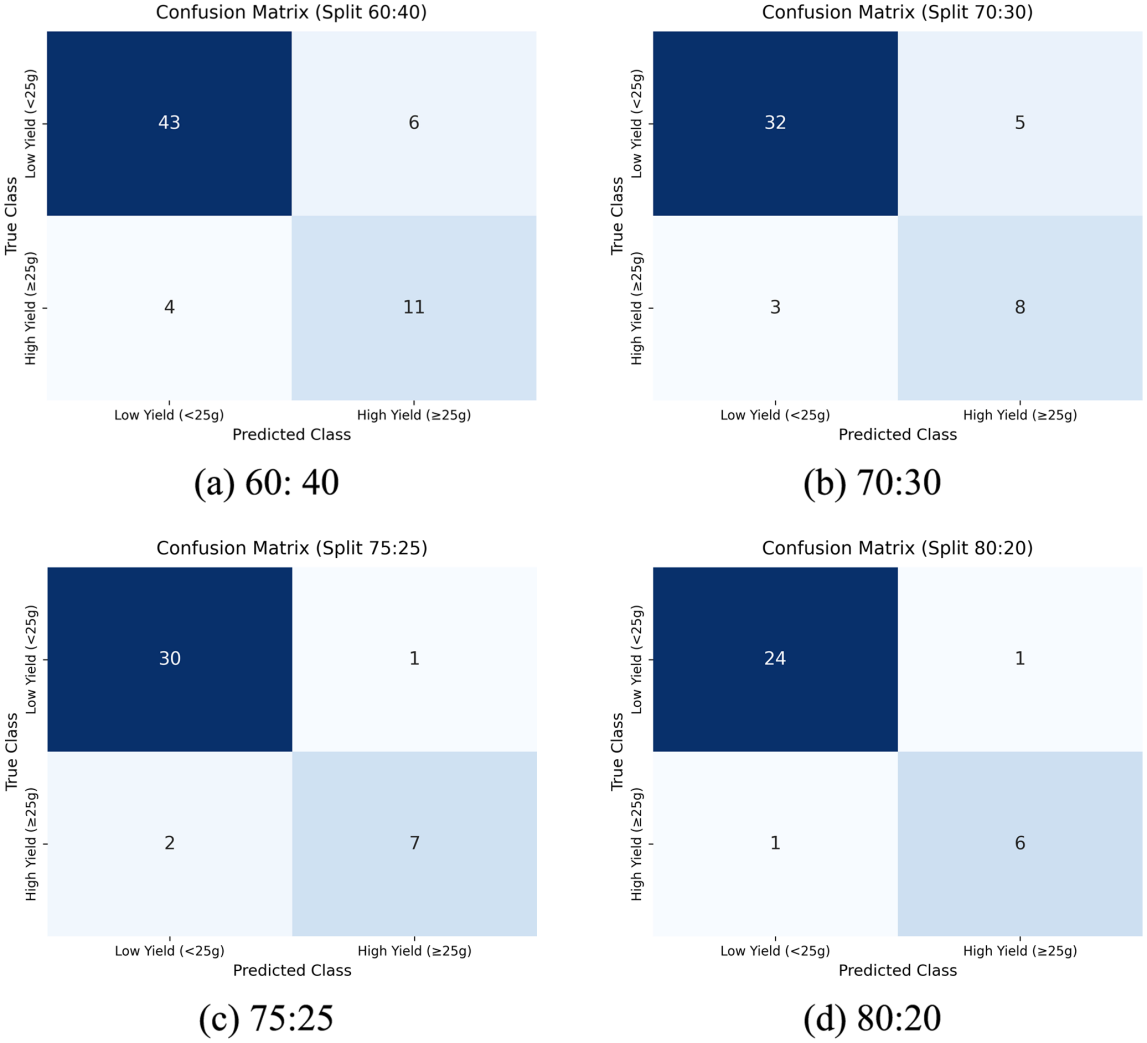
Confusion matrix of proposed test sizes.

**Figure 9:**
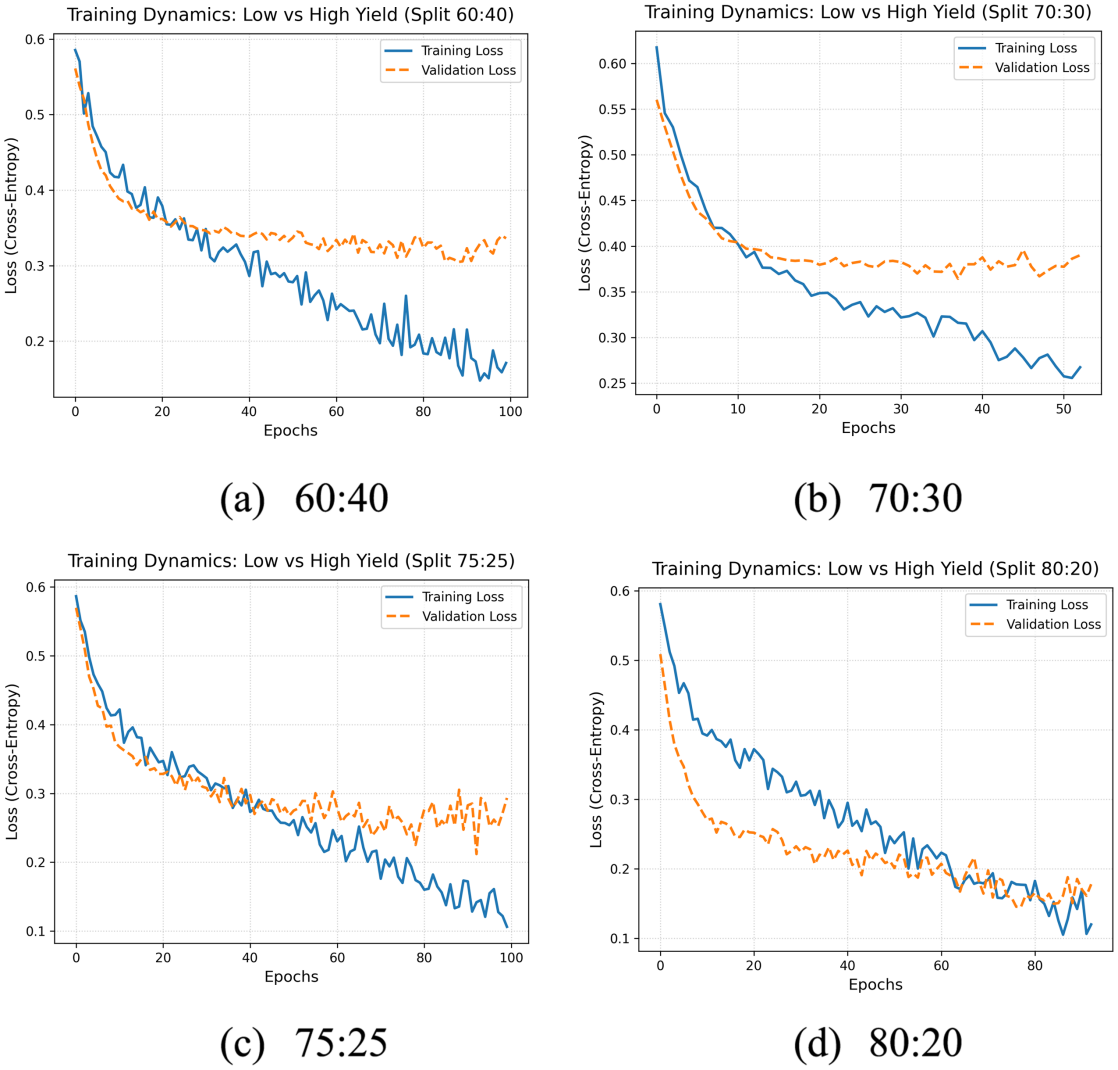
Training and validation loss curves of the proposed test size.

The initial search by HCA identified a configuration utilizing a single RBM layer (33 units), a dropout rate of 0.2, ReLU activation, Adam optimizer, a batch size of 2, and a learning rate 0.003 (Table 6). Training on the preferred 80:20 split achieved 0.97 accuracy on the training set and 0.94 on the testing set. Model performance was consistent across multiple train-test splits (60:40, 70:30, 75:25, 80:20), demonstrating robust classification with low error rates as summarized in Table 7. The confusion matrix and loss curves of these splits are presented in Figures 8 and 9, respectively, illustrating the clear separation between low and high yield classes.

**Table 6:** Optimal hyperparameters identified by HCA search.

Hyperparameters and accuracy	
RBM structure (hidden units)	[33]
Dropout rate	0.2
Activation function	ReLU
Optimizer	Adam
Batch size	2
Learning rate	0.003
Train accuracy	0.97
Test accuracy (80:20)	0.94

**Table 7:** Evaluation metrics values for varied train:test split configurations.

Metrics	60:40	70:30	75:25	80:20
Specificity	0.88	0.86	0.97	**0.96**
Error rate	0.16	0.17	0.07	**0.06**
Train	0.96	0.86	0.97	**0.96**
Test	0.84	0.83	0.93	**0.94**

Training accuracy (train), testing accuracy (test), error rate (ER).

#### 4.2.1 Final optimized configuration

While the initial HCA output yielded high accuracy, the small batch size (2) and moderate dropout (0.2) posed a potential risk of volatility on unseen data. Therefore, the model underwent a final fine-tuning step to enhance stability. The batch size was increased to 4 to stabilize gradient updates, and the dropout rate was increased to 0.30 to impose stricter regularization. This final configuration (Table 8) achieved a robust tes accuracy of 93.75% with an error rate of 0.06 and specificity of 0.96. This slight adjustment in parameters ensured that the model prioritized generalization over peak training performance.

**Table 8:** Fine-tuned hyperparameters

Hyperparameter	Value
RBM units	[33]
Dropout rate	0.30
Activation function	ReLU
Optimizer	Adam
Batch size	4
Learning rate	0.003

To address the limitations of simple accuracy metrics, we further evaluated the model using class-wise Precision, Recall, and F1-scores (Table 9). The model demonstrated exceptional performance in identifying class 0 (Low KW) with a precision of 0.96. Crucially, for the minority Class 1 (High KW), the model maintained a high precision and recall of 0.86, indicating that the HCA-DBN effectively learned to distinguish high-yielding ears without bias, despite the small sample size.

**Table 9:** Detailed class-wise performance metrics for the optimized HCA-DBN

Class	Precision	Recall	F1-score	Support
0 (low KW < 25g)	0.96	0.96	0.96	25
1 (High KW >= 25g)	0.86	0.86	0.86	7
Weighted average	0.94	0.94	0.94	32

**Figure 10:**
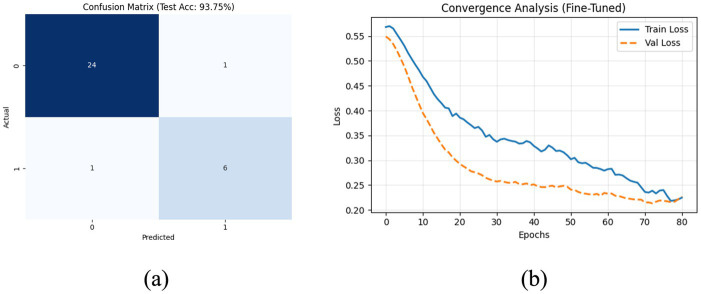
Performance visualizations post-optimization: **(a)** confusion matrix; **(b)** training and validation loss curves.

**Figure 11:**
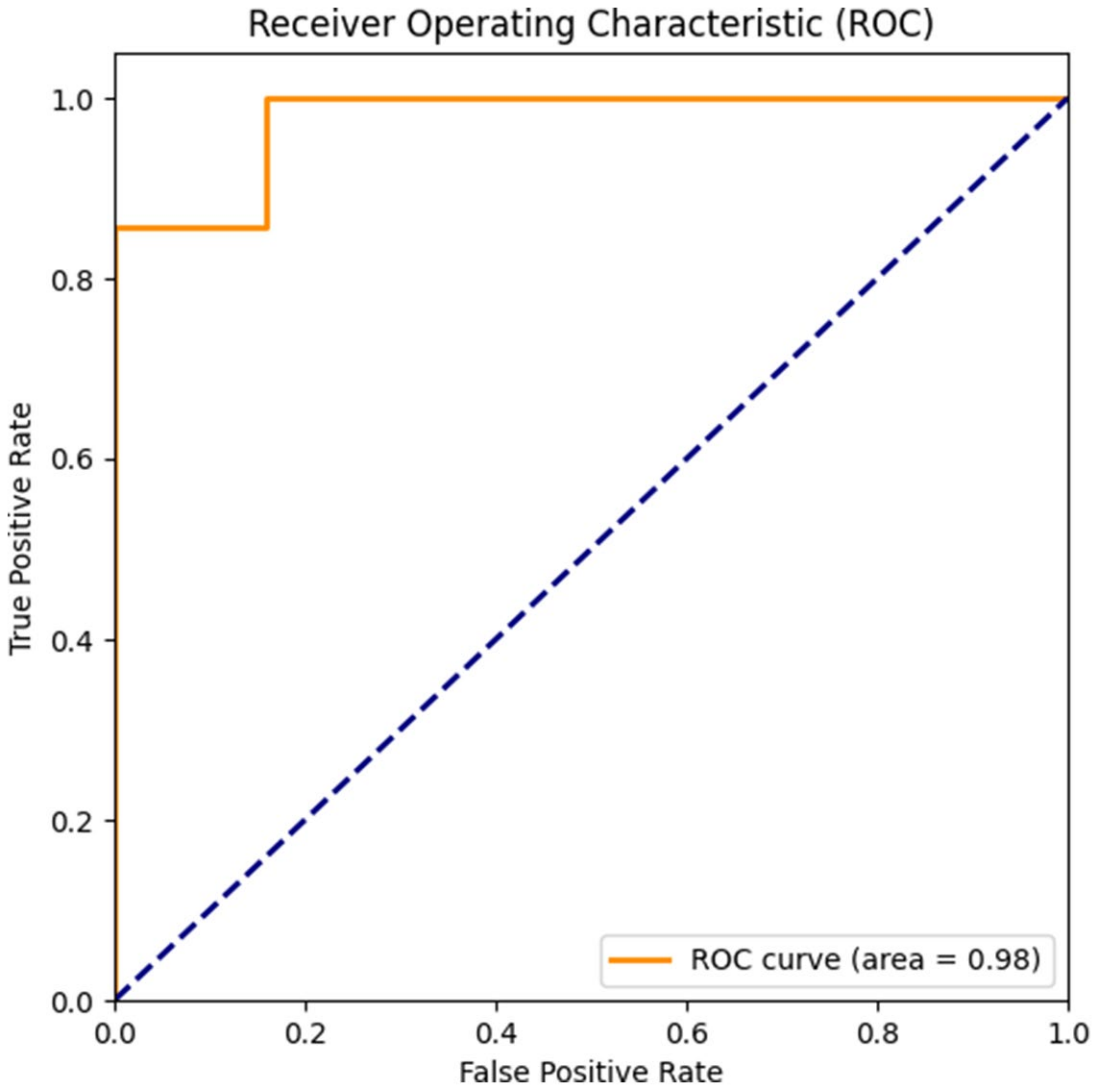
ROC curve for the optimized HCA-DBN model.

The final confusion matrix (Figure 10a) showed only 2 misclassifications out of 32 test samples, demonstrating high precision in identifying the target class. The stability of the learning process is further evidenced by the loss curves (Figure 10b), which show valid convergence without significant divergence between training and validation loss. The discriminatory power of the model is further visualized by the receiver operating characteristic (ROC) curve (Figure 11). The high area under the curve (AUC = 0.98) confirms that the classifier maintains a true positive rate while minimizing false alarms, reinforcing the robustness observed in the confusion matrix.

#### 4.2.2 Model robustness and stability analysis

Given the constraints of the dataset size, we performed additional validation to verify the model’s reliability beyond a single train-test split. A stratified 10-Fold Cross-Validation was conducted, yielding a mean accuracy of 83.63% ( ±0.10 ). While this is slightly lower than the peak accuracy of the best split, it represents a conservative and realistic estimate of the model’s generalizability. Additionally, Bootstrap validation (200 iterations) was performed to establish confidence intervals. The bootstrap mean accuracy was 88.79%, with a 95% confidence interval ranging from 0.73 to 1.00. These metrics statistically validate that the HCA-DBN framework captures meaningful patterns in the kernel weight data despite the limited sample size (Figure 10).

The HCA-DBN effectively classified individual ear kernel weight into low and high yield categories, consistently outperforming conventional classifiers. The transition to a simpler architecture (33 units) combined with rigorous cross-validation confirms that the model’s performance is robust and not merely an artifact of overfitting. These results highlight the model’s capability to capture complex interactions among plant and ear traits even with limited data (Figure 11).

#### 4.2.3 Feature sensitivity and biological interpretation

To address the high Variance Inflation Factors (VIF) observed in ear traits (Table 2) and investigate the impact of multicollinearity, a comparative study was conducted using dimensionality reduction and feature selection techniques. We evaluated the DBN’s configuration under four scenarios (i) all features, (ii) LASSO selection, (iii) PCA, and (iv) Mutual Information.

**Figure 12:**
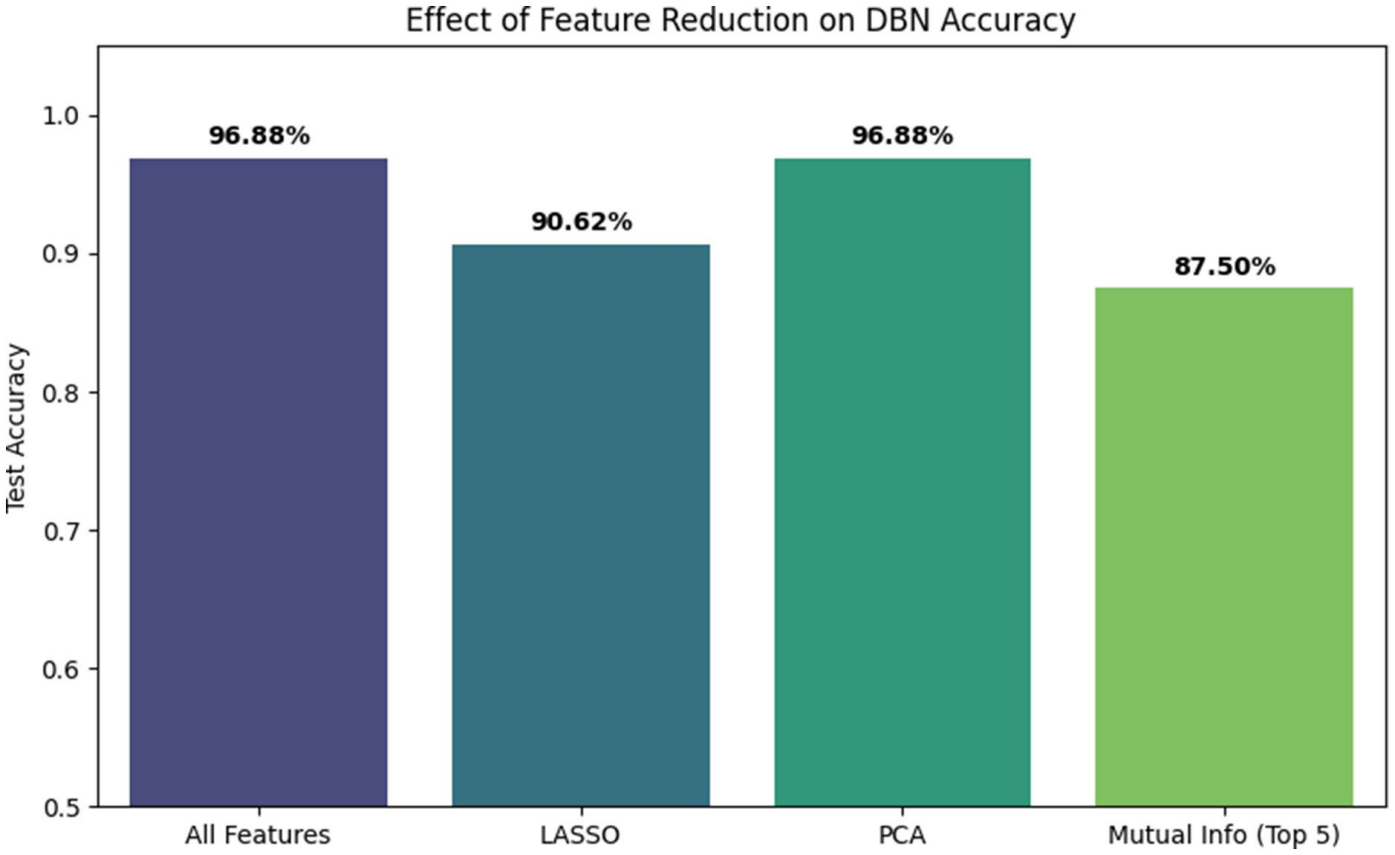
Effect of feature reduction techniques (LASSO, PCA, mutual information) on DBN classification accuracy compared to the full feature set.

As illustrated in Figure 12, the full feature set achieved the highest classification accuracy of 96.88%. Feature reduction via Mutual Information and LASSO led to performance degradation (dropping to 87.50 and 90.62%, respectively). This suggests that traits identified as collinear by linear metrics actually contribute unique biological information. For instance, the relationship between EWS and EWOS captures variability in husk density. Some plants produce heavy sheaths masking poor kernel set, while some exhibit thin sheaths with high grain filling. Retaining both features allows the deep network to disentangle these morphological subtleties, whereas aggressive feature selection removes these critical agronomic signals. While PCA maintained high accuracy, it offered no performance gain over the original feature space. Consequently, the complete feature set was retained to preserve all available morphological information for the hierarchical learning process.

**Figure 13:**
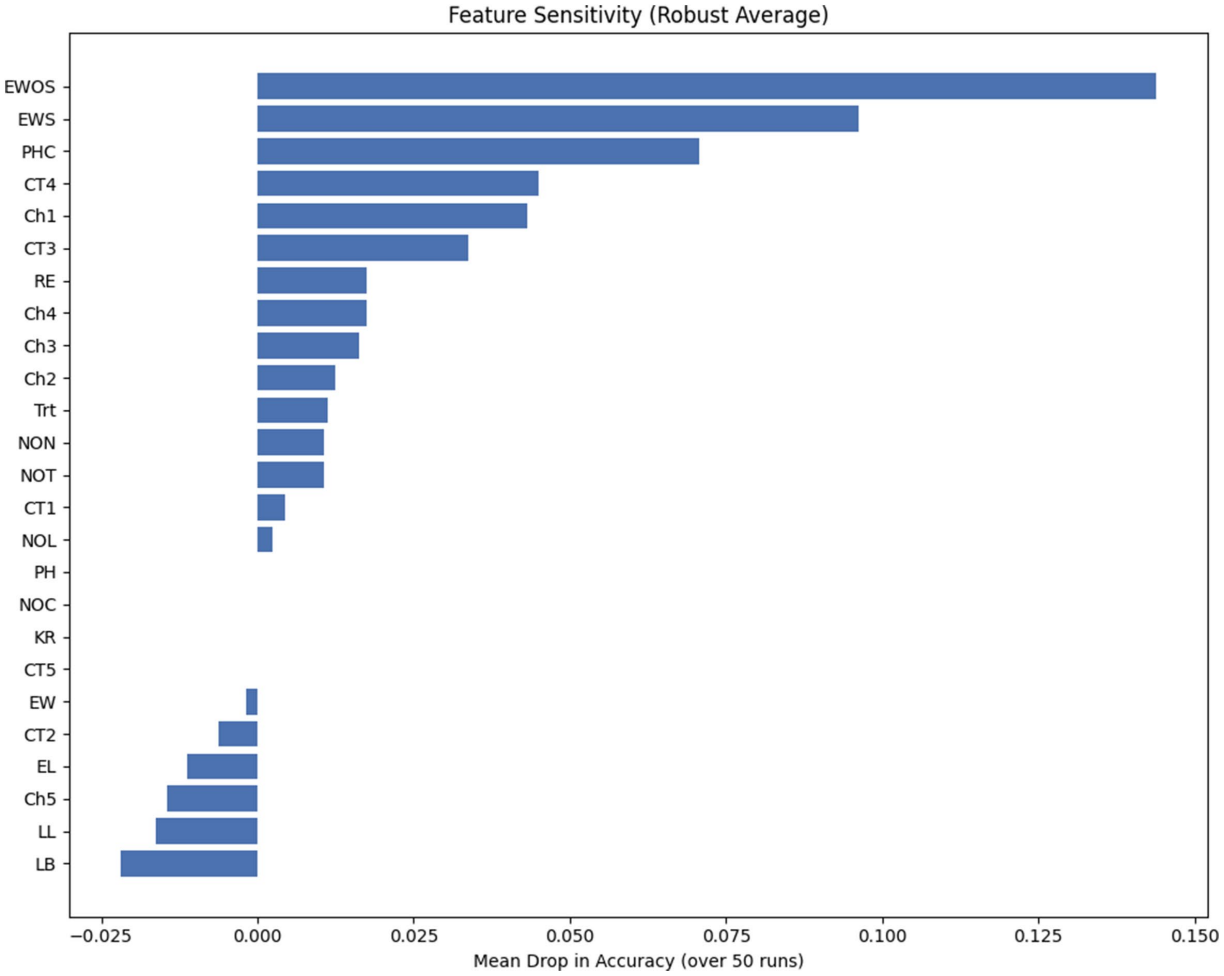
Robust feature sensitivity analysis.

To elucidate biological basis of the model’s predictive capability, a robust permutation-based sensitivity analysis was conducted, averaging feature importance scores over 50 stochastic iterations to ensure statistical reliability (Figure 13). The analysis demonstrates that the HCA-DBN classification inference is predominantly governed by reproductive morphology, with EWOS and EWS emerging as the principal determinants. This prioritization aligns with fundamental agronomic principles, identifying the biological sink capacity as the most direct proxy for yield. Furthermore, the high sensitivity ranking of both EWS and EWOS suggests the model effectively exploits the differential between these variables (husk biomass) as a critical indicator of yield potential.

Secondary architectural traits, particularly PHC, also exhibited significant influence, implicating the vertical stratification of the ear as a key factor in structural stability and nutrient translocation efficiency. Conversely, individual vegetative traits such as LL and LB displayed negligible inverse sensitivity indices. Far from suggesting biological irrelevance, this pattern underscores the architectural robustness of the HCA-DBN against multicollinearity. The model appears to abstract the source potential from the collective vegetative profile rather than relying on singular, noise-prone phenotypic traits. Consequently, when a specific vegetative feature is perturbed, the network compensates via correlated predictors, demonstrating a learned capacity to navigate high-dimensional morphological dependencies without requiring manual dimensionality reduction.

### 4.3 Comparison with alternative metaheuristic optimizers

To validate the efficiency of the chosen Hill Climbing approach, the DBN was optimized using five alternative metaheuristic algorithms: Particle Swarm Optimization (PSO), Genetic Algorithm (GA), Differential Evolution (DE), Grey Wolf Optimizer (GWO), and Whale Optimization Algorithm (WOA). As shown in Table 10, the HCA-DBN outperformed all complex metaheuristics. While GA achieved a moderate accuracy of 81.25%, it failed to match the 93.75% achieved by HCA. Other swarm-based methods (PSO, GWO, WOA) struggled to converge, yielding accuracies between 59 and 63%. Furthermore, Differential Evolution (DE) proved computationally expensive (65.7 s) without yielding performance gains. This suggests that for small sample datasets with high dimensionality, complex population-based heuristics may suffer from premature convergence or overfitting, whereas the local-search strategy of HCA efficiently navigates the optimization landscape to find a robust architecture.

**Table 10:** Performance comparison of HCA-DBN against standard metaheuristic optimization algorithms.

Optimization algorithm	Test accuracy	Hidden units found	Dropout	Time(s)
HCA (proposed)	**0.94**	**[33]**	**0.30**	**25.00**
GA	0.81	[88, 54]	0.09	36.92
DE	0.66	[16, 128]	0.00	65.74
PSO	0.63	[119,58]	0.33	36.57
WOA	0.63	[128, 128]	0.50	39.09
GWO	0.59	[128, 128]	0.50	36.96

### 4.4 Limitations of the study

While the proposed HCA-DBN model demonstrates superior performance, there are limitations to this study that must be acknowledged. First, the study utilized a relatively small dataset (*n* = 160), which may limit statistical power. To mitigate this, we employed rigorous validation techniques, including stratified 10-Fold Cross-Validation and Bootstrap analysis, to ensure the results are robust. Second, the current model classifies yield into binary categories (High vs. Low). While effective for initial screening, this simplifies the biological complexity of maize yield. Third, our model relies on morphological and physiological traits but excludes external environmental variables (e.g., rainfall and soil variation) which are also critical determinants of yield. Finally, as with most deep learning architectures, the DBN operates as black box; future iterations will focus on integrating Explainable AI (XAI) techniques to visualize feature importance.

## 5 Conclusion

This study presents a Hybrid Cascade - Deep Belief Network (HCA-DBN) framework for classifying maize kernel weight into low (< 25 g) and high (≥ 25 g) categories based on plant and ear traits. The model combines the hierarchical feature extraction capability of DBNs with Hill Climbing Algorithm-based hyperparameter optimisation to ensure efficient convergence without the computational cost of complex evolutionary methods. Comparative analysis with standard classifiers demonstrated that while MLPs achieved competitive accuracy, the HCA-DBN offered superior robustness and generalization, particularly for small and imbalanced datasets (Feng et al., 2017; Gopi and Karthikeyan, 2022). Although the study was limited by the dataset size (*n* = 160) and a restriction to a single organic growing season, rigorous validation techniques including bootstrapping (mean accuracy of 0.89) and stratified 10-fold cross-validation (mean accuracy of 0.84), confirmed the statistical stability of the results, ensuring that performance was not an artifact of train-test splits. Future research will address current limitations by validating this framework on larger, multi-season datasets to confirm generalizability across diverse agro-climatic zones. Key directions for future work includes extending the current binary classification to multi-class yield prediction (Low, medium, High) to provide more granular insights for farmers and integrating environmental variables, such as soil fertility and rainfall patterns, to create a holistic prediction system. The proposed HCA-DBN model ultimately establishes a reliable, data-driven benchmark for field-based maize kernel weight classification in resource-constrained agricultural settings.

## Data Availability

Publicly available datasets were analysed in this study. This data can be found here: https://doi.org/10.1016/j.dib.2024.110367.
